# Dental Patents

**Published:** 1858-11

**Authors:** John T. Toland


### DENTAL PATENTS.

The prevailing disposition to patent every thing in the Dental line, calls loudly for some united action of the profession to correct and counteract this restrictive mania, and it seems to us to be the duty of every man in the profession to set his back against it.

That Dentists are entitled to the same legal protection for useful inventions and improvements as any other member of community, we freely admit, and where the invention and improvement has real merit and value, we will go as far as any to encourage and sustain the patentee; the difficulty exists in patenting every new thought that inspires the mind of the seekers of notoriety, or in making some slight change in some previously established process without really affecting the principle involved, as well as in the mode of placing such improvements before the profession. As members of a liberal profession each one should be as ready to give as to receive, in all matters of operative Dentistry it is certainly asking nothing more than right, that those who have the benefits of the ideas, suggestions and experience of others, should in turn contribute their share to the general fund of information, and thus accumulate a literature alike beneficial to the profession and their patients.

Instruments, machinery, apparatus and materials may be patented with perfect propriety, and we feel confident that we reflect the sentiments of a majority of the profession when we state that there is no class, or profession more willing to pay for benefits received in improvements by which they are enabled to improve their operations, facilitate business and benefit their patients, because on this is dependent their own success; but so often has their confidence and liberality been imposed upon by designing men, who protected by a patent and supported by certificates, have canvassed the country with "a pig in a poke," to extort money from the unwary, who anxious to add to their stock of knowledge have paid their hard earned cash for what has, in most cases, proved to be no better than the "baseless fabric of a vision."

They have at last become like the burnt child who dreads the fire, and rather than be further imposed upon will either endeavor to evade the patent, or do without the proposed improvement.

There should be no antagonism between inventors and those who have use for the invention, as each is important to the other. Not one of all the Dental patents that have been issued that has ever been a source of profit to the owner, and for the simple reason that patentees have pursued a dictatorial and intolerant policy to which the profession would not submit, tamely, and to legally protect their claims, "costs more than it comes to."

If patentees would confine their claims to the articles themselves, and the manufacture and sale thereof at a reasonable price, no objection would be made, the profession could then have an opportunity to examine and judge of the value of the article, and purchase small amounts for experiment, and if not satisfactory, abandon it and return to first principles, without much loss, or if the article is really valuable, each can use what their practice demands, and thus pay in a just ratio to the benefits received.

We would suggest to all Dental associations, and especially to the "American Dental Convention," to take the matter in hand.

Let contributions be made, a sinking fund established, and an individual or committee appointed, to take charge of the fund, and whose duty it shall be to keep fully informed in regard to all Dental patents, from the time of issue until the claim is established, and investigate carefully the claims, both as it relates to practical utility and validity, and publish the results as soon as ascertained; and if after careful investigation it is ascertained that the patent is not valid, publish the fact for the information of the profession, and in the event of the patentee or owner of patent attempting to compel any member of the profession to pay tribute, or restrain him from using the process, it then becomes the duty of the individual or committee to take the case out of the hands of the individual Dentist, and with his superior information, at the expense of the association defend the suit at law. By such a course all parties would be benefitted.

The patentee or inventor who makes a *useful discovery* will have his improvement acknowledged and protected, and freely and promptly receive a liberal reward for time, labor and ingenuity, whilst on the other hand the profession will be protected from the demands and annoyances of spurious or worthless patents.

All of which is respectfully submitted to the discretion of wiser heads.

JOHN T. TOLAND.

